# Multiple Modelling Methods Suggest That Grain‐Free Diets Are Not Predictive of New Owner‐Reported Cardiac Diagnoses Over Two Years in the Dog Aging Project, Despite Owner Misclassification of Diet

**DOI:** 10.1002/vms3.71213

**Published:** 2026-09-16

**Authors:** Janice O'Brien, Morgan Bishop, Dog Aging Project Consortium, M. Katherine Tolbert, Audrey Ruple

**Affiliations:** ^1^ Virginia‐Maryland College of Veterinary Medicine Virginia Tech Blacksburg Virginia USA; ^2^ Gastrointestinal Laboratory, Department of Small Animal Clinical Sciences, College of Veterinary Medicine and Biomedical Sciences Texas A&M University College Station Texas USA

**Keywords:** cardiac, diet, dogs, grain‐free

## Abstract

**Background:**

Grain‐free diets have been potentially linked to dilated cardiomyopathy (DCM) in several studies in dogs, although the underlying mechanisms for this potential link, if present, are unclear. Additionally, some studies have found no association between grain‐free diets and DCM in dogs. Unfortunately, many of these studies were limited by the validity of owner‐reported diet information. Furthermore, the effect of grain‐free diets on other cardiac conditions in dogs is even less studied.

**Objectives:**

To assess the validity of owner‐reported grain‐free diet status information, and to model the potential effect of grain‐free diet exposures over the first several years of Dog Aging Project data collection.

**Methods:**

The validity of owner‐reported grain‐free status of the diet was evaluated by comparing owner‐reported grain‐free diet status to the ingredients. Several regression analyses were performed, accounting for likely confounding factors identified with a directed acyclic graph (DAG), to measure the effect of owner‐reported grain‐free diet status on all cardiac diagnoses. General linear mixed‐effects models and general linear regression models were used with similar results.

**Results:**

There was a large misclassification error in the diets reported to be grain‐free. In spite of this, modelling methods anticipate that any bias introduced into an effect estimate would be very small. Several modelling methods failed to find a statistically significant association between grain‐free status of the diet and any cardiac diagnosis. When including incident‐only cases in a linear model with confounders, the odds ratio for the effect of grain‐free diet status on any new cardiac diagnosis was 1.05 (0.91–1.21, *p* value 0.52), suggesting no effect.

**Conclusions:**

The findings suggested that the potential effect of grain‐free feeding on all owner‐reported new diagnoses of any cardiac disease within this dataset is likely minimal or absent.

## 1 Introduction

Grain‐free diets for companion dogs have attracted significant public interest and scientific scrutiny in recent years. These diets experienced rapid growth in US markets during the 2010s: at a chain brick‐and‐mortar boutique retailer, grain‐free diet sales began in 2011, increased threefold by 2015 and eventually comprised 43% of all extruded diets sold by that retailer in 2019 (Quest et al. 2022). Similar findings from 2017 found that grain‐free diets including peas, chickpeas, lentils, sweet potatoes and tapioca comprised 50.8% of the dog food market (Plantz 2017). In 2018, the US Food and Drug Administration (FDA) announced an investigation into reports correlating feeding of grain‐free diets with dilated cardiomyopathy (DCM) in dogs (Center for Veterinary Medicine 2020). Following this announcement, researchers across academia and industry initiated numerous studies to further investigate this potential link. Since the FDA's initial report, several studies have been published investigating this link using both randomized controlled trials and observational designs in populations of client‐owned and research colony dogs (Bagardi et al. 2022; Bakke et al. 2022; Banton et al. 2021; Banton et al. 2024; Bokshowan et al. 2023; Coppinger et al. 2024; Donadelli et al. 2020; Freeman et al. 2018; Gizzarelli et al. 2021; Haimovitz et al. 2022; Kaplan et al. 2018; L. Freeman et al. 2022; L. M. Adin et al., 2019, 2021; Leach et al. 2023; Owens et al. 2023; Quest et al. 2022; Quilliam et al., 2021, 2023; Singh et al. 2023; Smith et al., 2021, 2022; Verde et al. 2023; Walker et al. 2022).

Even though the FDA investigation and studies on grain‐free diets were focused on DCM (and associated mechanisms) specifically, and though no professional organization has made an official recommendation regarding grain‐free diets, many individual veterinarians began recommending avoiding grain‐free diets for any cardiac disease including murmurs and mitral valve disease out of an abundance of caution until more research could clarify the potential underlying mechanisms and true population at risk (Rausch 2024, VET Specialists n.d., Veterinary Hospital n.d.). Some also recommended avoiding grain‐free diets for dogs with a breed predisposition to any cardiac disease (Rausch 2024), again to be cautious while more research could be conducted on the topic. However, while the field of diet‐associated DCM has continued to have new research published, there has been a dearth of studies focusing on the effect of grain‐free diets and all cardiac disease.

Finally, and perhaps most important to consider in this context given the degree to which research on dog health relies on owner‐reported information, several studies have identified owner‐reported diet history data as a substantial methodological limitation. These limitations encompass both owner recall of the primary diet type itself as well as additional treats, supplements and food items (Walker et al. 2022; L. M. Coppinger et al. 2024; Freeman et al. 2018; Leach et al. 2023). Indeed, research investigating grain‐free diets and cardiac function has consistently found that owner reports may be overestimated, misattributed or incomplete. Consequently, researchers recommend that controlled, objective assessments provide more reliable data for evaluating dietary impacts on canine health (L. Freeman et al. 2022).

To address these limitations in the research field, and with the ultimate goal of providing valuable insight into the question of the extent to which diet influences cardiac health outcomes in companion dogs, we attempted to quantify the accuracy of owner‐reported data on their dog's diet in the Dog Aging Project (DAP). The DAP is an ongoing longitudinal study following more than 50,000 companion dogs by collecting data on dog health, lifestyle and environmental factors that contribute to dog health and longevity. The DAP collects diet information annually from dog owners with a companion dog enrolled in the study. From January 2020 to June 2023, this original diet survey asked basic questions about the dogs’ diets, including a question about whether or not the diet was grain‐free. It did not, however, collect detailed ingredient information about the diet until the comprehensive diet questionnaire was implemented in July 2023, which included survey items asking owners to provide details about the ingredients of the dogs’ diet and the name of the diet. The purpose of this study was (1) to evaluate the validity of owner reporting of grain‐free diet status in both diet survey versions to determine whether the first two years of DAP diet data can reliably be used in longitudinal studies and (2) conduct a longitudinal study with prospectively collected diet data, and apply Directed Acyclic Graph (DAG)‐guided confounder controls, with appropriate modelling methods to determine potential causal effect of grain‐free diets and all owner‐reported cardiac diagnoses within the DAP cohort.

## 2 Materials and Methods

### 2.1 Dog Aging Project Surveys

The DAP is a large, longitudinal and collaborative study following dogs of different breeds, ages, health and location. All dogs are permitted to enroll, which allows for a wide variety of participants: young and old dogs, and dogs of all breeds including mixed breeds. Dogs are enrolled once their owner completes an initial survey called the Health and Life Experiences Survey (HLES) (Dog Aging Project 2023), which asks many questions regarding the lifestyle, environmental exposures and health diagnoses of the dog. Once enrolled, some dogs are considered for inclusion in additional cohorts which also collect biological samples. Any dog can be a member of the ‘pack’, and owners are prompted annually to complete their Annual Follow Up Survey (AFUS) (Dog Aging Project 2023) which asks comprehensive questions about any changes that the dog has experienced within the last year (e.g., diet change, moving to a new state or house, etc.). The original diet survey, implemented from 1 January 2020 until 30 June 2023 collected data on the dog's primary diet component (51% or more of the diet), and whether the dog was fed more than one diet component, and what types of diets these were (e.g., kibble, canned, home‐prepared, etc.). The original diet survey also collected information regarding the dog's primary component's grain‐free status. In July 2023, in an effort to bolster data robustness, the comprehensive diet questionnaire was implemented. This survey was much longer than the original diet survey and collected more specific dietary data, including the full name of the primary diet component with the brand name. It also included a question which asked how long the owners had been feeding that diet (< 1 month, 1–3 months, 3–6 months, 6–9 months, 9–12 months or a year or more).

The HLES and AFUS surveys also collect owner‐reported health diagnoses data. A total of 336 health conditions are grouped into 19 health systems, and each year owners are asked to report whether any new diagnoses have occurred. The cardiac system disease options are: aortic/subaortic stenosis, atrial septal defects, mitral dysplasia, murmur, patent ductus arteriosus (PDA), persistent right aortic arch, pulmonic stenosis, tricuspid dysplasia, ventricular septal defects, arrhythmia, cardiomyopathy, congestive heart failure, endocarditis, hypertension, pericardial effusion, pulmonary hypertension, subaortic stenosis, valve disease and other cardiac condition (which is a write‐in free text option). Owners can report multiple new disease diagnoses within the same category in the same year. These new diagnoses are then reported within HLES and AFUS surveys with their condition code, and the month and year of the reported diagnosis. If an enrolled dog dies or is euthanized, owners are prompted to complete the End of Life Survey (EOLS) (Dog Aging Project 2023) which collects information about new health diagnoses since the time of the last AFUS completion.

The data analysed for this study were extracted from the 2023 DAP curated data release (Terra n.d.). The University of Washington IRB deemed that recruitment of dog owners for the DAP, and the administration and content of the DAP Health and Life Experience Survey (HLES), are human subjects research that qualifies for Category 2 exempt status (IRB ID no. 5988, effective 10/30/2018). No interactions between researchers and privately owned dogs occurred; therefore, IACUC oversight was not required.

### 2.2 Grain‐Free Diet Status Validation

**FIGURE 1:**
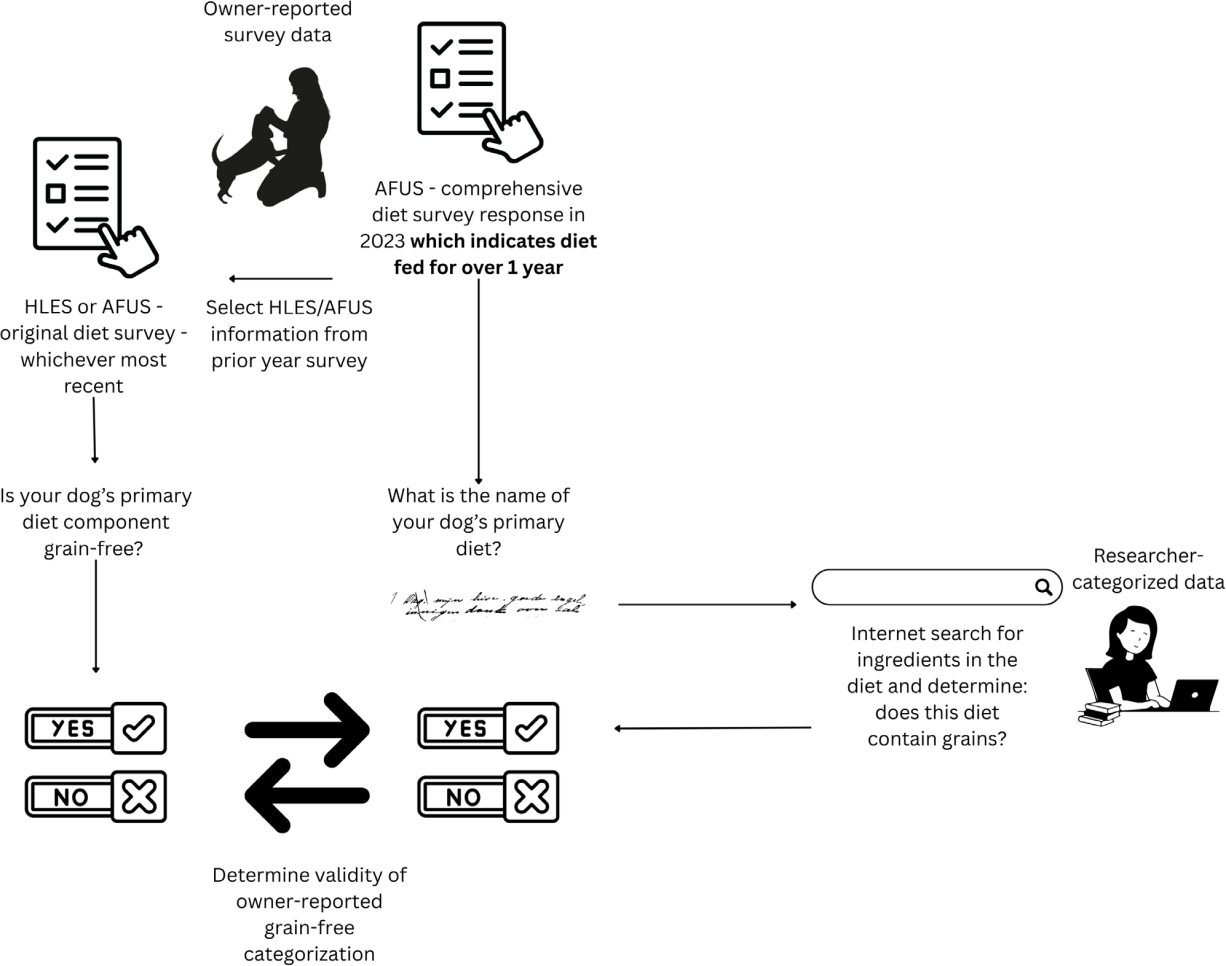
Process of data selection for validation of owner‐reported grain‐free status of diets in the Dog Aging Project, 2023.

DAP participants who had completed an AFUS in 2023 that contained the comprehensive diet questionnaire questions, and had reported that they had been feeding the same diet for at least 12 months were selected for inclusion in this study. Their previous year answers were extracted from either the previous year's AFUS or HLES survey responses, whichever was the most recent. This meant that the same diet was reported on for the yes/no grain‐free status of the diet under the original diet survey, while getting more detailed brand and diet name information from the comprehensive diet survey, so that the two were directly comparable (see Figure 1).

Owner responses for the grain‐free status of their pet's diet on the original diet survey, and the name of the diet reportedly fed for over 12 months on the comprehensive diet questionnaire were compiled into an Excel spreadsheet with data organized into columns including brand name/formula, duration fed, if the primary diet component was reportedly grain‐free, and if the primary diet component was actually grain‐free. Each response was individually validated against ingredient lists found online in April 2025 for each brand name and formula by a single coder (M.B.). The brand name and formula were searched using a web‐based search engine. If the brand's website was available, ingredient lists were checked there first; if not, a third‐party website containing ingredient information for the dog food brand was used. After the dog food ingredient list was found, it was reviewed to identify the presence of different types of grains including rice, corn, wheat, barley and oats. If the dog food ingredient list contained one or more grains at any location in the ingredient listing, it was classified as grain‐inclusive, and if it did not contain any grains, it was classified as grain‐free. In a case where the owner's response did not specify a brand name or formula type and a specific dog food ingredient list could not be found, ‘cannot determine’ was coded. This process was repeated for each response until the spreadsheet was completed.

The owner‐reported grain‐free status and the attributed grain‐free status as coded manually were then compared to each other. Diets for which ingredients could not be determined were excluded, and the overall accuracy, positive predictive value (PPV) and negative predictive value (NPV) were calculated using the R package caret (Kuhn 2008), and a sankey plot was created using the R package ggplot2 (Wickham 2009).

### 2.3 Simulations of Effect Estimations Given the Misclassification Error

A simulation study was conducted to examine how owner misclassification of dietary exposure could bias the observed association between a grain‐free diet and a binary health outcome. In each simulation, a synthetic population of 10,000 dogs was used, where a proportion (0.4) was assigned to a grain‐free (GF) diet to represent the 40% of dogs reportedly consuming a grain‐free diet as represented in this dataset. The probability of the health outcome (e.g., cardiac disease) was assigned to different risk levels, with these risks varying across simulation conditions to represent different true effect sizes: protective (10% risk in GF, 30% risk in GI), none (10% risk for both GF and GI), mild (15% risk in GF, 10% risk in GI), moderate (30% risk in GF, 10% risk in GI) and severe (50% risk in GF, 10% risk in GI).

To simulate misclassification, a reporting inaccuracy was introduced such that only a given proportion (which varied between 25%, 50% and 75%) of grain‐free dogs were correctly reported as such. All grain‐inclusive diets were assumed to be correctly reported. This generated a second, ‘reported’ exposure variable that reflects what might be collected via owner self‐report. Then, 2 × 2 contingency tables were calculated for both the actual and reported diet exposures versus the health outcome, and odds ratios were calculated using the package epitools (Aragon 2020).

Simulations were repeated 200 times for each effect size combination and misclassification rate (25%, 50% and 75%) using the package purrr (Functional Programming Tools [R package purrr version 1.1.0] 2025). The resulting dataset captured the distribution of true versus observed odds ratios under each misclassification and effect size scenario, allowing for evaluation of the magnitude and direction of bias introduced by diet misclassification. These results were plotted using ggplot2.

### 2.4 Estimation of the Effect of Grain‐Free Diet Status on Cardiac Diagnoses Using a General Linear Logistic Regression Model With the First Three Years of Diet Data

HLES and AFUS original diet questionnaire data regarding the grain‐free status of the primary diet component were extracted for each dog for each survey year as applicable. Developed (i.e., not present at birth) cardiac condition diagnoses were also extracted for each dog for each survey year from HLES and AFUS. No cardiac conditions were excluded. Dogs could have more than one cardiac condition reported, and having a condition reported at HLES did not prevent a new one from being reported at AFUS (e.g., if a dog had a murmur reported at HLES and then developed congestive heart failure on an AFUS, the new cardiac condition was still included). Using EOLS, all new owner‐reported cardiac diagnoses (including but not limited to those that contributed to the passing of the dog) were captured, and the most recent diet information for grain‐free diet was used (last observation carried forward) from the most recently completed survey (HLES or AFUS) prior to the dog's death.

Year of participation in the study was included as a factor in the linear model to reduce any potential differences between surveys and as a proxy for age. Two logistic regression models were fit: one base without any covariates and one adding covariates for breed status (single‐breed vs. mixed‐breed), sex, neuter status, size (weight range in 10 lb bins) and survey year (i.e., the year of the dog's participation in the survey). These confounders were identified using a DAG (see the Supporting Information).

### 2.5 Other Modelling Methods

Mixed‐effects models and a diet‐switching analysis were also performed. The mixed‐effects model was fit first since it is considered the standard for causal analyses of prospective data. However, the mixed‐effects model produced wide confidence intervals and became unstable when confounders were added. For this reason, the general linear model (logistic) regression was chosen as the primary analytic approach. The methods and results for these models can be found in the Supporting Information.

## 3 Results

A total of 47,444 dogs were enrolled in DAP with an HLES completed prior to 31 December 2023. Of these dogs, 26,842 completed at least one AFUS. EOLS surveys for 7786 dogs were completed by the same date.

### 3.1 Validation of Owner‐Reported Grain‐Free Status of the Primary Diet Component

**FIGURE 2:**
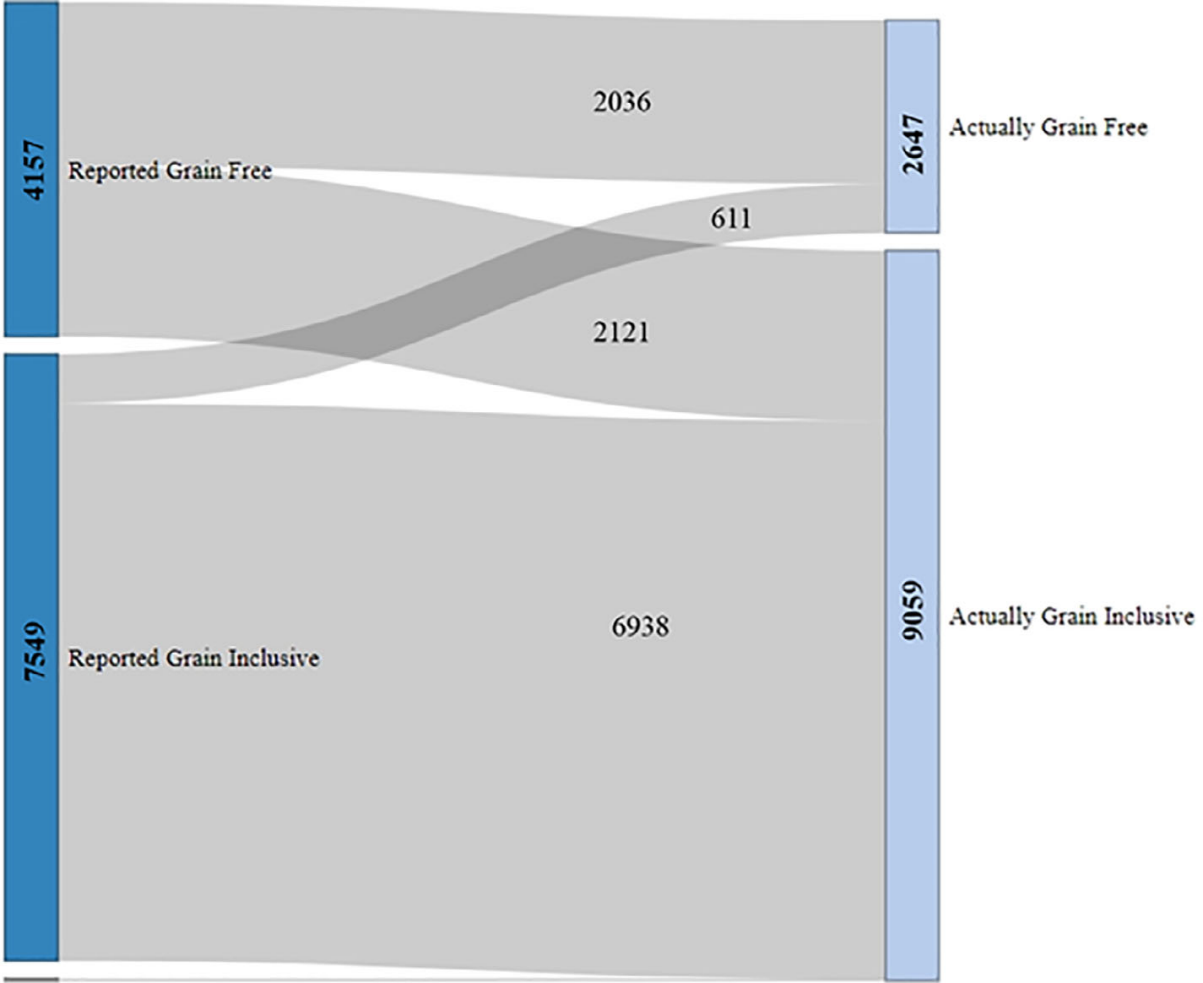
Sankey diagram of owner‐reported grain‐free and grain‐inclusive status of 13,049 dog diets, and the actual grain‐free and grain‐inclusive status of those diets based on ingredient listing.

A total of 13,049 individuals had completed AFUS surveys which included a response to the comprehensive diet questionnaire and also a previous response to the initial diet survey, and had indicated that the diet on the comprehensive survey was fed for over 1 year duration. A total of 35% of responses reported dogs to be fed grain‐free diets. The PPV, or the likelihood that the owner's reporting of their dog's food being grain‐free was correct, was 49%. The NPV, or the likelihood that the owner's reporting of their dog's food being grain‐inclusive was correct, was 92%. The overall accuracy was 77%. For a sankey diagram of these results, see Figure 2.

### 3.2 Simulations of Effect Estimations

**FIGURE 3:**
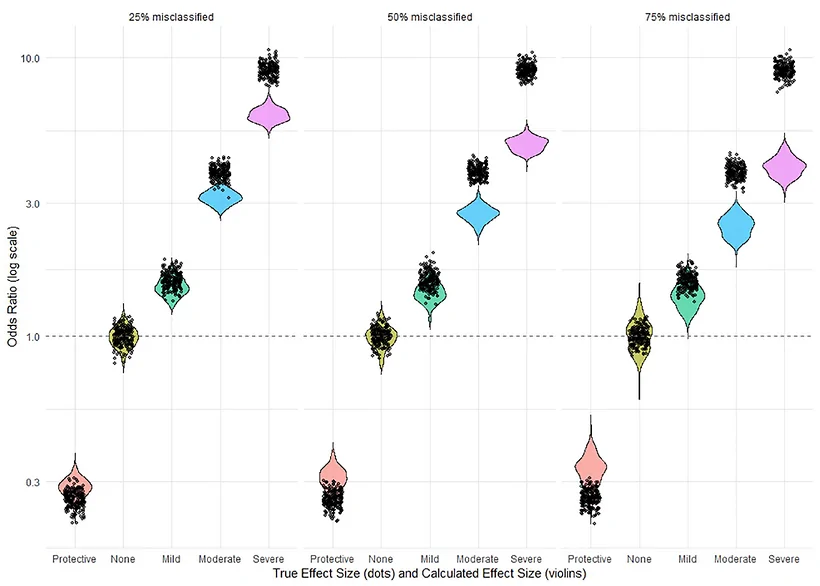
Observed and true effect size estimators of various levels of misclassification bias across several effect sizes.

Despite the misclassification determined from the validation data, simulations of this particular misclassification show that computed effect estimates would tend closer to an odds ratio of 1 compared to the true effect (i.e., the magnitude of the computed association would be diminished compared to the true association), but would not create a false association (i.e., the direction of the association would not be impacted). So, in spite of large misclassification bias, this particular type of bias would not introduce false effects, but would instead marginally diminish true effects. Measures indicating an absence of correlation (odds ratio 1) would not be changed. See Figure 3.

### 3.3 Estimation of the Causal Effect of Grain‐Free Diet Status on Cardiac Diagnoses Using General Linear Models With the First Three Years of Diet Data

A total of 47,444 unique dogs were included in this analysis, including 7786 dogs for which EOLS had been completed, 550 of which had a reported cardiac condition at the time of death which had not been reported in HLES or AFUS (see Table 1). Among dogs who had a cardiac diagnosis reported in AFUS, the most common diagnoses were murmur (57%), congestive heart failure (9%), valve disease (9%) and arrhythmia (8%) (see Table 2).

**TABLE 1:** Grain status of diets and developed cardiac conditions in Dog Aging Project participants using the original (non‐comprehensive) diet survey data. Dog Aging Project, 2023.

Survey data	Grain‐free diet	Grain‐inclusive diet	No developed cardiac condition	Developed cardiac condition
HLES (year 0)	24,085	16,244	44,784	2660
AFUS (year 1)	10,674	6408	8404	531
AFUS (year 2)	334^a^	168^a^	5115	418
EOLS	3113^b^	2299^b^	7236	550

^a^ Only 502 dogs had responses for the original diet survey on AFUS year 2. The remainder completed the comprehensive diet questionnaire.

^b^ Diet data for EOLS were used (last observation carried forward) from the most recent prior survey data. Please note that not all dogs had diet information reported.

**TABLE 2:** All cardiac diagnoses reported for dogs with developed cardiac conditions who also had responses to the original diet questionnaire as part of the AFUS dataset in the Dog Aging Project.

Cardiac diagnosis	# of dogs
Murmur	521
Congestive heart failure	81
Valve disease	79
Arrhythmia	77
Cardiomyopathy	54
Hypertension (high blood pressure)	50
Other cardiac^a^	23
Pulmonary hypertension	12
Subaortic stenosis^b^	6
Pericardial effusion	5
Aortic/Subaortic stenosis^b^	4
Mitral dysplasia	3
Endocarditis	3
Patent ductus arteriosus (PDA)^b^	2
Pulmonic stenosis^b^	2

^a^ Other cardiac includes: cardiomegaly (14), stroke, syncope, heart blockage and chordae tendineae rupture. Note: some dogs have more than one reported diagnosis within the cardiac disease category.

^b^ These conditions are noted to be congenital (or early developmental) conditions that were reported for dogs with a different developed condition reported at the same time. This was seen to be a likely possibility, given that perhaps dogs with congenital conditions only receive a diagnosis after developing clinical signs for a developed condition such as congestive heart failure, and at that point both conditions are diagnosed on cardiac ultrasound. These dogs were not excluded for having an existing congenital condition, given that part of the discussion surrounding grain‐free diets is that they may worsen cardiac function for dogs with pre‐existing conditions and hasten the development of secondary disease.

The unadjusted odds ratio for the effect of grain‐free diet on cardiac outcomes was 0.98 (0.91–1.05, *p* value 0.56). When adjusted for confounders, the adjusted odds ratio for the effect of grain‐free diets on new cardiac diagnoses was 1.05 (0.91–1.21, *p* value 0.52), suggesting no effect of grain‐free status on risk of new cardiac diagnoses. While not part of our primary aims, we present odds ratios for all covariates included in the adjusted models to enhance transparency and allow readers to evaluate the potential influence of these variables on the outcome. Covariates which were associated with new cardiac diagnoses were: year (new diagnoses more likely later in the study—OR 1.20, 1.03–1.38), breed status (single‐breed dogs more likely compared to mixed‐breed dogs—OR 1.23, 1.06–1.42), neuter status (with neutered animals more likely to be diagnosed compared to intact ones—OR 1.68, 1.2–2.43) and size (with larger weight ranges having smaller odds ratios). Sex did not correlate with new diagnoses (OR 0.89, 0.77–1.02) (for full covariate results, see the Supporting Information).

## 4 Discussion

Regarding the accuracy of owner reporting of grain‐free status of their dog's diet, the NPV was much higher than the PPV, suggesting that owners are more accurate at identifying grain‐inclusive diets compared to grain‐free diets. When taken in context with previous studies, this suggests that owner‐reported diet data on the grain status of their dog's diet is likely biased, with owners reporting grain‐free diets more likely to be incorrect than those reporting grain‐inclusive diets. The reasons for this are unknown, as DAP surveys pertaining to diet and health do not collect information about owner intentions or motivations when it comes to feeding choices. Based on impressions from coding the individual diet names, diets incorrectly categorized by owners as being grain‐free that were actually grain‐inclusive commonly did not have a grain listed in the title (e.g., ‘Brand name Meat and Potato recipe’ or simply ‘Brand name life stage’) while having a grain in the ingredients list. Because of this, it is likely that individual grains are differently impacted by this misclassification since different grains appear in diet titles. Therefore, it is possible that this misclassification bias is driven by naming conventions of diets.

Although we found a relatively large degree of inaccurately reported grain‐free diets in this population, our simulations show that this type of error would mostly reduce the strength of true associations rather than create false ones. In other words, computed odds ratios would appear closer to ‘no effect’ (OR = 1) than they actually are, but a protective effect would not be observed if the true association was harmful, and vice‐versa. Based on the degree of attenuation observed in the 50% misclassification simulation, we estimate that an ‘estimated’ odds ratio of 1.05 would represent a true odds ratio around 1.07. This is useful information to consider when attempting to use both the original diet survey data and the comprehensive diet questionnaire data in future longitudinal studies: the data from the original diet survey can be used regarding grain‐free diet status, as it will result only in attenuated associations. This finding is also perhaps reassuring to previous researchers in this field, suggesting that owner‐reported diet data on this particular diet subject most likely did not meaningfully impact the strength or direction (positive or negative) of the reported associations. This does assume that the misclassification present in the DAP population is similar to any misclassification present in other studies. Previous studies of grain‐free diets vary in their diet classification methods, and especially in their reporting of the methods which makes direct comparison difficult. Most studies report investigator classification of owner‐reported diet information from retrospective medical records (Adin et al. 2019; Freid et al. 2021; Walker et al. 2022) without fully describing whether the owners reported grain‐free status or a full diet name. Some studies do detail that ingredient‐level information was classified by investigators (L. Freeman et al. 2022) or that brand information was available (Ontiveros et al. 2020).

In interpreting the findings regarding the validity of owner‐reported grain‐free diet data, there are limitations that must be considered. The first one is recall bias—it is possible that owners reported feeding the same diet for over one year when in fact they had switched diets. However, it is not expected that this recall bias would be differential between owners feeding grain‐free and grain‐inclusive diets. Additionally, the diet names were reported on the surveys in 2023, but the ingredients research was conducted in 2025, so it is possible that the diet reported was reformulated and contained different ingredients at the time it was fed. Additionally, the reliability of the owner‐reported cardiac diagnoses data is unknown. Electronic health record validation for certain disease categories has been completed with DAP data (Schmid et al. 2026), and while cardiac conditions were not one of the target disease categories investigated in detail, 10 of 20 disease categories had agreement over 90%, with the highest agreement categories being diseases for which further testing is necessary for confirmation (endocrine, and immune conditions were over 99%), while those with more disagreement were conditions which owners could observe (oral and dermatologic conditions). Given that all cardiac conditions require at least a veterinarian to diagnose (if not a cardiologist), this suggests that owner‐reported cardiac conditions are a reliable source of data at the category level (if not at the individual condition level).

By using prospectively collected diet and cardiac diagnosis data, controlling for confounders identified using a DAG, and using several modelling approaches, this analysis moves beyond purely descriptive associations to be more consistent with causal inference. The estimate for the effect of grain‐free diet status on incident cardiac diagnoses, when controlling for appropriate demographic variables and considering dogs which have passed, shows no effect between grain‐free diet status and owner‐reported cardiac health diagnoses in the DAP population. This finding was consistently observed using several analytical methods. Based on all these analyses, there is very likely no effect of grain‐free diet status on all owner‐reported cardiac diagnoses in this population. Time (calendar year), dog size, dog neuter status (independent of age), and breed status were more associated with cardiac diagnoses. Given that neuter status had the strongest association with cardiac disease diagnoses, it should be strongly considered in any future analyses of diet and cardiac disease, and the extent to which the absence of accounting for it in any previous studies has impacted the results from those investigations. There are multiple reasons for why neuter status may be the most strongly associated demographic variable—it may be biological (perhaps sex hormones have a protective effect on the cardiovascular system in dogs), or it may be because neutered dogs live longer and therefore have more time at risk. While time was used in the model, it represented participation time in the DAP cohort, and perhaps age at diagnosis would be a more accurate factor to include for future analyses.

One important consideration when interpreting these analyses is that we considered only the incident diagnosis of all owner‐reported cardiac outcomes. In this population, the most common cardiac diagnoses were valvular disease or suspected valvular disease (indicated by the presence of a heart murmur). So, while this is preliminarily reassuring information for practitioners who have been recommending transitioning dogs with valve disease away from grain‐free diets as a precautionary measure, this particular study would likely not be sensitive enough to detect the effect of grain‐free diets on the incidence of individual cardiac diseases, nor does it investigate the effect of diet on the survival of dogs with individual diseases. Future studies investigating whether the survival of dogs with diagnosed valvular disease is impacted by diet would add important insights to our body of knowledge (and are currently underway). This analysis is preliminarily reassuring, however, that grain‐free diet status does not impact incident diagnoses of cardiac conditions as a whole.

The most important limitation to this study is that individual ingredients could not be considered in the longitudinal analysis, since this information was not collected in the original DAP diet survey. As previous research has reported, individual ingredients contained within grain‐free diets may be causing echocardiographic changes associated with DCM and not all grain‐free diets contain these ingredients. The ingredients most frequently associated with DCM cases in previous studies as potentially being linked to DCM when fed in high proportions are potatoes and pulses such as lentils and peas, with some studies indicating that peas may pose greater concern than lentils or potatoes (Quilliam et al. 2023). One study found no short‐term differences in blood taurine concentrations between dogs fed grain‐free versus grain‐inclusive diets, and it is known that taurine deficiency can lead to DCM. Nevertheless, the authors concluded that the amino acid digestibility was impaired in the high‐pulse diets, potentially putting dogs at risk for taurine deficiency and, therefore, diet‐associated DCM during long‐term feeding (Quilliam et al. 2021).

The data collection period (2020–‐2023) coincides with a critical timeframe in the grain‐free diet controversy. By this period, the potential link between grain‐free diets and DCM risk had been widely publicized for several years, creating substantial pressure and incentive for reformulation. Many manufacturers producing grain‐free diets likely modified their formulations during or prior to this study period, potentially removing or reducing ingredients suspected of contributing to diet‐associated DCM. This temporal factor may explain why this study failed to detect associations between grain‐free diets and cardiac diagnoses. If industry reformulation has indeed addressed the problematic ingredients, this would represent a positive development for pet owners who prefer grain‐free options. However, this hypothesis underscores the importance of continued monitoring of commercial diet ingredient information in longitudinal studies, as it suggests that the safety profile of grain‐free diets may be formulation‐specific rather than categorically determined by grain status alone.

Despite inherent limitations, this study possesses several notable strengths. The extended duration of dietary exposure and prospective nature of the data collection provide robust methodological foundations, particularly when combined with inclusion of data captured at the time of a dog's death. By including dogs who passed during the study period, this design accounts for dogs who developed sudden illness leading to death. However, sudden death events lacking diagnostic confirmation may have been miscategorized, which represents a particular concern for cardiac‐related mortality where definitive diagnosis often requires post‐mortem examination.

## 5 Conclusion

This study failed to find a positive or negative effect of owner‐reported grain‐free diets on new cardiac diagnoses using many different modelling techniques. While it is possible that a small effect may be present, but attenuated. Even though grain‐free diets were not accurately reported, the particular way in which the inaccuracy occurred meant that any meaningful effect should have been observed if present. This is preliminarily reassuring information for pet owners and veterinary practitioners who have been avoiding all grain‐free diets for any cardiac disease. However, to fully determine which diets have any impact on individual cardiac diseases, considerably more research needs to be done.

## Author Contributions

**Dog Aging Project**: writing – review and editing. **Audrey Ruple**: funding acquisition, conceptualization, methodology, supervision, writing – original draft, writing – review and editing, project administration, resources. **Morgan Bishop**: investigation, data curation, writing – original draft, writing – review and editing. **Janice O'brien**: investigation, conceptualization, data curation, formal analysis, methodology, writing – original draft, writing – review and editing, visualization, validation. **M. Katherine Tolbert**: supervision, writing – original draft, writing – review and editing.

## Funding

This research is based on publicly available data collected by the Dog Aging Project, which is supported by U19 grant AG057377 from the National Institute on Aging, a part of the National Institutes of Health, and by additional grants and private donations, including generous support from the Glenn Foundation for Medical Research, the Tiny Foundation Fund at Myriad Canada, and the WoodNext Foundation. This specific project was supported through an NIH T32 grant for researcher development.

## Ethic Statement

The University of Washington IRB deemed that recruitment of dog owners for the Dog Aging Project, and the administration and content of the DAP Health and Life Experience Survey (HLES), are human subjects research that qualifies for Category 2 exempt status (IRB ID no. 5988, effective 10/30/2018). No interactions between researchers and privately owned dogs occurred; therefore, IACUC oversight was not required.

## Conflicts of Interest

M. Katherine Tolbert received research grants from the Comparative Gastroenterology Society, Morris Animal Foundation, Winn Feline Foundation, and The ACVIM Foundation. Audrey Ruple received grants and lecture honoraria from Morris Animal Foundation and Purina Institute. The remaining authors declare no conflicts of interest.

## Supporting information




**Supplemental Figure 1**: Directed acyclic graph used to identify likely confounding factors and their relationship to the exposure of diet and the outcome of cardiac diagnoses.
**Supplemental Figure 2**: Odds ratio effect estimates with 95% confidence intervals for switching analysis to estimate the effect of diet exposure on new cardiac diagnoses within the Dog Aging Project pack 2020–2023.
**Supplemental Table 1**: Linear mixed effects model with confounders added in a step‐wise manner: only the effect estimate for grain free diet status is reported for each model.
**Supplemental Table 2**: Effect estimates for grain‐free diet status and several identified confounding factors included as covariates in a general linear (logistic) regression model for 40,329 dogs in the Dog Aging Project, 2020–2023.
**Supplemental Table 3**: Observed and true effect size estimators of various levels of misclassification bias across several effect sizes; as calculated with calculated effect sizes closer to odds ratios less than 1.5.

## Data Availability

These data are housed on the Terra platform at the Broad Institute of MIT and Harvard.
