# Supplementary material for: Multiple Modelling Methods Suggest That Grain‐Free Diets Are Not Predictive of New Owner‐Reported Cardiac Diagnoses Over Two Years in the Dog Aging Project, Despite Owner Misclassification of Diet
Source: Vet Med Sci. 2026 Sep 16;12(5):e71213. doi: 10.1002/vms3.71213 (PMC13581454; doi:10.1002/vms3.71213)
Supplement: Supplementary file 1 [file VMS3-12-e71213-s001.docx]

**Supplemental material**

**Supplemental figure 1:** Directed acyclic graph used to identify likely confounding factors and their relationship to the exposure of diet and the outcome of cardiac diagnoses.

*Linear models for effects of grain-free diets over first two years of diet survey:*

To account for repeated measures over time within individual dogs, a generalized linear mixed effects model (GLMM) was used, with the glmer function from the lme4 package in R. This model included a random intercept for each dog, allowing within-dog correlations across time points to be considered. Fixed effects included diet type (grain-free vs grain-inclusive), time, and their interaction to evaluate whether the effect of diet type on cardiac outcome varied over time. Only AFUS cardiac diagnoses were used to assess whether grain-free diet predicts onset of new cardiac disease. Only dogs with an HLES and at least one AFUS were included, so this analysis excluded dogs with HLES-only data.

The logistic linear mixed effects model in R (glmer) with both dog and owner demographic variables failed to converge and had large standard errors for many predictors. The dog weight in pounds was scaled. However, this did not resolve the convergence issue. Therefore, a step-wise method to building the model was taken, starting with the diet exposure term alone and the scaled weight of the dog. Then the following demographic terms were added to a simple logistic regression model in a stepwise manner: sex, neuter/spay status, breed status, and weight. The estimate for grain free diet exposure was tracked for each step to understand how it changed with the addition of each covariate.

$$log((\frac{P\left( {Cardiac}_{it} \right)}{1-P\left( {Cardiac}_{it} \right)}= \beta_{0}+\beta_{1}{GrainFree}_{it}+\beta_{2}{Time}_{it}+\beta_{3}\left( {GrainFree}_{it} \times{Time}_{it} \right)+ \beta_{4}{Weight}_{i}+ \mu_{i}$$

Where:

- $P({Cardiac}_{it})$ is the probability of a cardiac condition for dog *i* at time *t*
- ${GrainFree}_{it}$ is the binary status of dog *i*’s diet’s grain-free status at time *t*
- ${Weight}_{i}$ is the dog’s weight (scaled)
- $\mu_{i}$ is a random intercept for dog *i*

**Supplemental table 1:** Linear mixed effects model with confounders added in a step-wise manner: only the effect estimate for grain free diet status is reported for each model

| Step | Estimate of grain free status | 95% CI low | 95% CI high |
| --- | --- | --- | --- |
| Base model (GF only) | 0.9557 | 0.2562 | 3.565 |
| GF+sex | 0.9482 | 0.2541 | 3.539 |
| GF+sex+neuter | 1.8972 | 1.8963 | 1.898 |
| GF+sex+neuter+breed | 0.9306 | 0.2495 | 3.471 |
| GF+sex+neuter+breed+age | 0.9179 | 0.2458 | 3.427 |

This dataset included 14,226 datapoints (2 time points for 7113 individual dogs), and 878 cardiac diagnoses (from the AFUS data). Initially, it was attempted to evaluate the relationship between a grain-free diet and cardiac disease using a logistic linear mixed-effects model, incorporating repeated measures for each dog across two time points. While the dataset was large (7113 unique dogs), the model failed to converge and produced unusually large standard errors for many predictors.

The base linear model which only accounted for weight and the interaction effect for diet with time was not significant (see **Supplememtal Table 1**). When adding in demographic variables as potential confounders in a step-wise manner, the effect estimate for the grain-free status of the diet did not change significantly, except when both sex and spay were added. This particular step also had an unrealistically small standard error, and likely demonstrates collinearity between the sex and neuter status variables. The effect estimate remained small and statistically non-significant regardless of which covariates were included, with consistently wide confidence intervals (see **supplemental table 1**)

*Diet-switching analysis*

Given the lack of interaction between diet and time in the mixed-effects model, the impact of diet was subsequently assessed using general linear models as a logistic regression with no random effects. The exposure was reframed to assess whether a dog had "never," "started," "stopped," or "always" been on a grain-free diet over a two-year period from 2020-2023. By treating dietary changes as categorical predictors, this analysis attempted to isolate the relationship between diet change and risk of cardiac disease. Time was not included as a term in this model since it was inherent in the “change” variable.

$$log((\frac{P\left( {Cardiac} \right)}{1-P\left( {Cardiac} \right)}= \beta_{0}+\beta_{1}GrainFreeSwitch+\beta_{2}Sex+\beta_{3}SpayNeuter+\beta_{4}Weight+\beta_{5}BreedStatus$$

The point estimates and 95% confidence intervals were computed and graphed using ggplot2 (see **Supplemental figure 2**).

**Supplemental figure 2**: Odds ratio effect estimates with 95% confidence intervals for switching analysis to estimate the effect of diet exposure on new cardiac diagnoses within the Dog Aging Project pack 2020-2023

This analysis utilized the same 7113 dogs from the previous analysis: dogs with one HLES and at least one AFUS with the old diet survey data. Dogs that started a grain-free diet had a lower odds for developing a cardiac condition compared to dogs that never ate a grain-free diet. Dogs that stopped or were always on a grain-free diet had no statistically significant difference for cardiac diagnosis compared to dogs that never ate a grain-free diet in this time period.

**Supplemental table 2:** Effect estimates for grain-free diet status and several identified confounding factors included as covariates in a general linear (logistic) regression model for 40,329 dogs in the Dog Aging Project, 2020-2023.

|  | Term | Odds ratio | p-value | 95% CI low | 95% CI high |
| --- | --- | --- | --- | --- | --- |
|  | Diet component grain free | 1.05 | 0.52 | 0.91 | 1.21 |
|  | Study year 2 | 1.21 | 0.48 | 0.69 | 1.99 |
|  | Study year end of life | 1.20 | 0.02 | 1.03 | 1.38 |
|  | Breed Status: Single-breed | 1.23 | 0.01 | 1.06 | 1.42 |
|  | Sex: Female | 0.89 | 0.10 | 0.77 | 1.02 |
|  | Weight (lbs): 11-20 | 0.61 | 0.00 | 0.48 | 0.79 |
|  | 21-30 | 0.45 | 0.00 | 0.34 | 0.60 |
|  | 31-40 | 0.23 | 0.00 | 0.16 | 0.33 |
|  | 41-50 | 0.26 | 0.00 | 0.19 | 0.36 |
|  | 51-60 | 0.17 | 0.00 | 0.12 | 0.24 |
|  | 61-70 | 0.16 | 0.00 | 0.12 | 0.22 |
|  | 71-80 | 0.15 | 0.00 | 0.10 | 0.21 |
|  | 81-90 | 0.12 | 0.00 | 0.07 | 0.19 |
|  | 91-100 lbs | 0.15 | 0.00 | 0.08 | 0.24 |
|  | 100+ lbs | 0.15 | 0.00 | 0.09 | 0.24 |
|  | Neutered | 1.68 | 0.00 | 1.20 | 2.43 |

**Supplemental table 3:** Observed and true effect size estimators of various levels of misclassification bias across several effect sizes; as calculated with calculated effect sizes closer to odds ratios less than 1.5

| Effect size model | Misclassification | Mean true OR | Mean calculated OR |
| --- | --- | --- | --- |
| None | 25% misclassified | 1 | 1.008 |
| None | 50% misclassified | 1.005 | 1.003 |
| None | 75% misclassified | 0.995 | 0.988 |
| Mild | 25% misclassified | 1.054 | 1.044 |
| Mild | 50% misclassified | 1.06 | 1.045 |
| Mild | 75% misclassified | 1.056 | 1.034 |
| Moderate | 25% misclassified | 1.117 | 1.103 |
| Moderate | 50% misclassified | 1.118 | 1.085 |
| Moderate | 75% misclassified | 1.117 | 1.085 |
| Severe | 25% misclassified | 1.604 | 1.486 |
| Severe | 50% misclassified | 1.602 | 1.407 |
| Severe | 75% misclassified | 1.592 | 1.339 |
